# Situating the KTA gap in clinical research: Foregrounding a discontinuity in practices

**DOI:** 10.3389/fpsyg.2022.1058845

**Published:** 2023-01-13

**Authors:** Giulia Di Rienzo

**Affiliations:** Centre for Philosophical Psychology, Department of Philosophy, Universiteit Antwerpen, Antwerpen, Belgium

**Keywords:** knowledge-to-action gap, scientific practices, ecological psychology, enactivism, knowledge translation, Science and Technology Studies (STS)

## Abstract

In this study, I will claim that we need to rearticulate the so-called “knowledge-to-action” (KTA) gap metaphor in clinical research as a discontinuity of practices. In clinical research, there is a significant delay between the production of research results and their application in policy and practice. These difficulties are normally conceptualized through the metaphor of the KTA gap between scientific knowledge and practical applications. I will advise that it is important to reformulate the terms of the problem, as they suggest the difficulty lies only in the results generated on one side (the laboratory), not reaching the other side (the clinic), and that crossing the gap requires us to simply optimize the transfer and exchange of knowledge. This perspective considers knowledge separate from the practices from which it was generated, making it into a thing that can be transported and transferred largely independently from the communities that produce or “possess” it. The paper then revises the terms of the problem, shifting the focus from knowledge understood as independent from practical circumstances to the situated practices of knowing. Knowledge will then be understood as enacted in practice, emerging as people interact recurrently in the context of established practices. When people coming from different domains and with different “ends-in-view” must coordinate, they have to deal with conceptual and practical tensions, different ways of doing things with their surroundings, and different normative practices. Considering that, the KTA gap will be revised, not as a gap between scientific results and their application in clinical practice, but as a discontinuity in how communities engage with their local contexts and what they perceive as relevant for their activities.

## 1. Introduction

For the past 20 years, studies in “evidence-based medicine” (EBM) have been popular in clinical research. The EBM movement claims that the best way to innovate is to apply new scientific results to medical practice or policy-making (D’Andreta et al., 2013). However, it has been observed that the enormous resources being put into biomedical research as well as the developments in understanding disease mechanisms are not resulting in proportionate improvements in new treatments, diagnostics, and prevention (Butler, 2008). Moreover, there is a considerable delay between the production of research evidence to improve health and wellbeing and its application to policy and practice. In the clinical sector, this time lag is estimated to be around 17 years^1^ (Balas and Boren, 2000; Institute of Medicine (US) Committee on Quality of Health Care in America, 2001; Morris et al., 2011). The difficulties have been conceptualized as a “knowledge-to-action” (KTA) gap between scientific results and practical applications (Rynes et al., 2001; Rushmer et al., 2019).

A well-known phenomenon is, for example, that doctors and practitioners do not apply guidelines.^2^ Nowadays, clinicians are asked to follow guidelines and instructions, grounded in scientific evidence, on the “best practice” to perform in a certain situation (Baiardini et al., 2009). Modern clinical guidelines aim to identify, summarize, and evaluate the most current data about prevention, diagnosis, prognosis, therapy, risk/benefit, and cost-effectiveness (Thomas et al., 1999) and to “develop(ed) statements to assist practitioners’ decisions about appropriate healthcare for specific clinical circumstances” (Institute of Medicine (US) Committee to Advise the Public Health Service on Clinical Practice Guidelines, 1990). However, although numerous efforts have been made to develop and disseminate evidence-based guidelines, practitioners seem not to apply them.

An example of a KTA gap could be found in the case study conducted by Newell et al. (2003), which will be covered more extensively in the Section 2” From the KTA gap to knowledge translation”. They highlighted how the knowledge generated by a new “best practice” in the diagnosis and treatment of cataracts, which was successfully designed through the interactions of various healthcare specialists, once it was transferred to other hospitals, was dismissed as unworkable.

However, the KTA gap is a relatively recent problem. Back in the 1950s and 1960s, basic and clinical studies were inextricably linked, as practically medical research was mostly done by physicians, i.e., scientists who also treated patients. In the 1970s, with the increased popularity of molecular biology, clinical and basic research started to separate and differentiate into several disciplines, with their own training and career paths (Butler, 2008). This separation created several communities, each pursuing different goals and led by different norms and concerns (Restifo and Phelan, 2011), which contributed to making any interchange between them problematic.

The historical context for the gap in clinical research is more than just anecdotal, as it allows us to appreciate the material issues from which the problem emerged. Its history tells us that practices of medicine and science, previously coupled together, evolved into separate practices, which became part of new patterns of customs and behaviors enacted in different working environments and by different trained experts. By contrast, the metaphor of a KTA gap suggests that the problem lies only in the “knowledge” generated on one side (the laboratory), which fails to reach the other side (the clinic), and that crossing the gap requires us to optimize the transfer and exchange of knowledge. This perspective considers knowledge independent of practical circumstances, transforming knowledge itself into a thing that can be handled, reproduced, stored, and transferred largely independently from the communal practices in which it is produced. This common articulation of the problem constrains the types of solutions formulated, limiting them to create knowledge infrastructures and knowledge management models to lead knowledge from one side of the gap to the other, and backgrounding all the contextual factors, such as the differences among communities, on both sides of the “gap.”

The aim of this study was to rearticulate the terms of the problem, shifting focus from knowledge understood as independent from practical circumstances to the situated practices of knowing. Knowledge will then be understood as enacted in practice, emerging as people interact recurrently in the context of established practices, and within their local situations. Considering that, the KTA gap will be revised not as a gap between scientific results and clinical practice but as a discontinuity in the way communities engage with their local context and in what they actually perceive as relevant for their activities.

## 2. From the KTA gap to knowledge translation

The metaphor of the KTA gap is a straightforward way to articulate the problem of coordination between basic science and clinical practice: there is a gap between the empirical results and their practical application.

As for the strategies to actually cross the gap, the relevant literature is often so varied that it is difficult to identify a coherent and explicit position (Graham et al., 2006). Undoubtedly contributing to the confusion in the area is the use of multiple terms, which belong to different conceptual backgrounds, to describe all or part of the process. For example, Ian Graham et al. (2006) identified 29 terms used to refer to some aspects of the concept of knowledge to action: “Some of the more common terms applied to the KTA process are knowledge translation, knowledge transfer, knowledge exchange, research utilization, implementation, dissemination, and diffusion” (Graham et al., 2006). Furthermore, some of these terms, such as “knowledge transfer,” are also used in research fields outside of healthcare. Generally speaking, in fact, “knowledge transfer” is used to indicate the process of transferring some type of knowledge to the relevant stakeholders, and that could be, in principle, applicable to many research fields, such as knowledge management and organizational studies.

Nevertheless, it is possible to see a “shared attitude” in articulating strategies to bridge the gap. With “shared attitude,” it is not meant to be something coherent and monolithic as an explicit framework but rather a tendency to organize solutions. A good example of this shared attitude is one of the two strategies employed by the Midlands NHS Trust, described in the case study conducted by Newell et al. (2003).

### 2.1. Bridging the gap: An attempt

The aim of the study conducted by Newell et al. was to understand the processes involved in the development and transfer of new best practices in cataract diagnosis. A core assumption in knowledge management is that the successful transfer of “best practices” is fundamental, as it prevents organizations from reinventing the wheel, especially in clinical environments. But Newell et al.’s findings challenge this logic, suggesting that generating knowledge about current practice is a precursor to changing those practices.

At the time, the British government targeted cataract diagnosis and treatment as an area that needed innovation due to the long-term nature of the diagnostic process. Typically, patients begin their journey at the optometrist “because they believe that deteriorating eyesight suggests the need for new glasses” (Newell et al., 2003). Since optometrists are not medical doctors, they are licensed to practice just primary vision care. Thus, when the optometrist diagnoses that the problem is cataracts, she refers the patient to her general practitioner. This contributes to making the patient’s journey very complex, because the general practitioner, not being an eye specialist, normally relies on the diagnosis made by the optometrist and refers the patient to the ophthalmologist, a doctor authorized to perform surgery. The ophthalmologist finally confirms the diagnosis and puts the patient on the waiting list after a few more examinations.

Due to the complexity and dispersion of the cataract diagnostic procedure, the UK government decided to involve several experts from the Midlands NHS Trust Hospital to re-engineer the entire procedure, simplifying and delivering a guideline for the best practice to be transferred to other NHS Trust hospitals.

The authors of the case study contrasted two processes that they observed during their investigation: the dynamic and cooperative process of successfully generating and applying the new best practice locally, and the process of transferring the newly developed guidelines to the other hospitals.

The first process, which involved the reorganization of cataract diagnosis and treatment, was highly successful. The authors attributed their success to the cooperative and joint sense-making processes experienced by the project participants. To reorganize the practice, participants had to build meaning from conflicting and confusing data, but more importantly, they had to coordinate their different opinions and needs, their tensions, and their different expertise. Through this constant exchange of ideas and skills, through continuous negotiations, all of the professional groups not only started to recognize each other’s skills and expertise, but they were also able to develop and apply the new practice.

In the newly developed procedure, optometrists were given additional responsibilities and training to decide from the very beginning of the patient’s journey whether she needed cataract surgery. In fact, the optometrists had to fill out a document that provided the consultant with very specific information about the patient and her cataracts, so that the ophthalmologist could arrange the operation. This eliminated non-essential visits to the GP, ophthalmologist, and other specialists, making the process leaner and faster. In fact, lead times were reduced from more than 12 months to between 6 and 8 weeks, significantly improving patient satisfaction.

However, in the second process, the attempts to transfer this “best practice” to other NHS hospitals have not been successful. One of the results of the project was the detailed diagnostic form that the optometrist had to fill out and send to the ophthalmologist. Once this form was developed, together with the descriptions of the new best practice, it was made available to other NHS trust hospitals, which, however, dismissed them as unworkable in their context.

The reasons that have been given for the ineffectiveness of knowledge transfer are varied. For example, one hospital that had considered the “best practice” rejected it because it was seen as “too radical.” Changes to an existing practice were said to take considerable time and effort, especially considering the workload of some working groups involved. In fact, “in many cases, consultants are keen to change things, but they feel that the clinical load is so great that they just get on and work to the best of their ability within the current system (Project Manager)” (Newell et al., 2003). It was also reported that most people in Midlands NHS Trust Hospital’ project shared a strong motivation to change existing practice and dovetailed a particular leadership style; this created a “receptive” context for change (for more details, refer to Pettigrew et al., 1992). But in the other trusts, where this new “best practice” could have been potentially equally relevant, the context may be much less receptive. This last process of knowledge transfer of best practice has a long tradition. It is a perspective that sees the application of knowledge as in principle separated from its generation, and that it is enough to make a new practice available to ensure that it will be implemented. This strategy of thinking about ways to bridge the KTA gap is called knowledge translation.

Knowledge translation (KT) has been defined first in 2000 by the Canadian Institutes of Health Research (CIHR) and then, at a consensus meeting of the World Health Organization in 2005, as “the synthesis, exchange, and application of knowledge by relevant stakeholders to accelerate the benefits of global and local innovation in strengthening health systems and advancing people’s health.” Because the problem is understood as a gap between empirical results and their practical applications, the alleged solution is the translation of these empirical results into clinical practice. For these reasons, researchers focused on developing knowledge infrastructures or knowledge management models aimed at bringing new scientific evidence to the clinical world.

A useful example of how KT is generally thought of is the global model developed in 2005 by the CIHR (Canadian Institute of Health Research):

KT1: Defining research questions and methodologies.
KT2: Conducting research (as in the case of participatory research).
KT3: Publishing research findings in plain language and accessible formats.
KT4: Placing research findings in the context of other knowledge and sociocultural norms.
KT5: Making decisions and taking action informed by research findings.
KT6: Influencing subsequent rounds of research based on the impacts of knowledge use (Canadian Institutes of Health Research Knowledge Translation).^3^

This is a general version of the same strategy used by the Midlands NHS Trust when it came to transferring its product to other clinics. The CIHR model approach represents a standard articulation of the steps to take to shift knowledge from its place of origin to clinical practice. As it is common, it returns in various forms in the plethora of KT models developed over the last 20 years.^4^

There is a large and ongoing debate on the effectiveness of KT models (Bero et al., 1998; Grimshaw et al., 2001; Sudsawad, 2007), but it is generally underlined that the benefits are modest. Models that normally use interventions that actively address specific barriers to change are said to be more effective, but they are hardly generalizable. In addition, it is difficult to distinguish which elements of such interventions have led to success. For this reason, the literature concludes that one of the problems to be solved is that there is not enough evidence to support decisions about which KT strategy is likely to be effective under different circumstances (Sudsawad, 2007).

### 2.2. Unpacking the KTA gap metaphor: Unquestioned assumptions

Although there are many ways to discuss knowledge translation, they all maintain similar characteristics. This is because they are all based on the same way of understanding the problem, which is that the issue to be solved is the gap between knowledge and application. The terms of the problem have never been fundamentally questioned, and this means that the way of shaping the solutions is constrained by the terms of the problem itself. This lack of questioning means that we may not be able to find an effective solution because we are looking at the problem in the wrong way. In fact, articulating the problem of coordination between basic science and clinical practice as a KTA gap means defining possible solutions around certain unquestioned assumptions that have been underlined and criticized by several authors (see Cook and Brown, 1999; Brown and Duguid, 2001; Greenhalgh and Russell, 2009). The similarities between the assumptions made about the KTA gap, and the strategies used to overcome it through knowledge translation, can be seen by observing again the Canadian Institutes of Health Research [CIHR] (2005) model.

First, the generation of knowledge is seen as clearly detached from practice, with theory and practice being two distinct phases (Yakhlef and Rietveld, 2020). The CIHR model suggests that the generative phase (research) and the implementation phase (clinical practice) are separated and connected only by the “placement” of research evidence in KT4, where the model suggests “placing research findings in the context of other knowledge and sociocultural norms” and with KT6 through “influencing subsequent rounds of research, based on the impacts of knowledge use.” In other words, there is no direct connection between research and clinical practice, but only an indirect connection through evidence placement and knowledge use. Knowledge is considered a product of scientific practice that, once produced, can be detached from the scientists who generate it and the practitioners who may use it (Greenhalgh and Wieringa, 2011). Consider, as an example, the traditional view on innovation (Rogers, 1962/2003). Innovation is divided into a “generative” phase of knowledge creation or discovery and an “implementation” phase, where that novelty is put into action; these are understood as two temporally and sequentially distinct phases (Anderson et al., 2014; Yakhlef and Rietveld, 2020), because the knowledge produced by the “generative phase” is expected to guide action in the “implementation phase.”

Second, because the empirical results of the scientific process and the process itself are detached, knowledge is treated as an object (see Greenhalgh and Wieringa, 2011; Bolisani et al., 2012). As an object, knowledge is thought to be a thing that can be handled, reproduced, stored, and transferred largely independently from the individual that produces or possesses it (Bolisani et al., 2012). In other words, knowledge is “reified”: it is turned into a thing. This thing is then often treated as if it were the underlying source of the process in which it was produced (see Dewey, 1896; Giddens, 1979; Heft, 2001; Ingold, 2011; Winther, 2014; Van Dijk and Myin, 2018. For a more in-depth discussion, see van Dijk, 2016. As knowledge as an object becomes more important, the practical processes from which it came are backgrounded. The sociocultural norms, the skills required, and the tensions among the communities are all seen as nothing more than “background information” or “context.”

This brings us to understand why the most popular strategies to cross the gap were formulated as the displacement of knowledge from one context to another. In this case, the KT4 step is emblematic. As clearly stated, it is necessary “to situate research findings” within the context of other knowledge and sociocultural norms. However, the “context” or “sociocultural norms” often seem to be treated as a container in which evidence can be “placed.”

**FIGURE 1:**
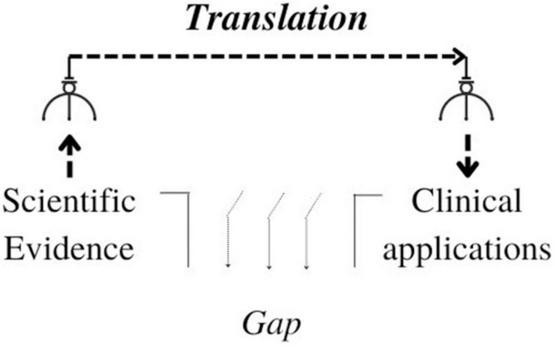
Knowledge translation (KT) as a claw machine.

If we follow this line of reasoning, it means we are constraining the strategies of KT into a sort of “claw-machine” metaphor (Figure 1).

The input is a scientific result, which is produced by basic science, while the output is the practical use of this evidence in the clinical setting. Translational research–in this frame–is understood as a claw, leading empirical evidence from its site of origin to application in clinical organizations.

The claw-machine metaphor is useful for understanding the KT model proposed by the CIHR and helps us visualize how the KTA gap could shape KT as a field. For this reason, I claim the KTA gap is unable to foreground the practical processes in which knowledge is enacted, as it will be shown in the following Section “3 From knowledge as an object to knowing in practice”.

## 3. From knowledge as an object to knowing in practice

If we focus our attention on knowledge as an object, something that can be simply moved from one part of the gap to the other, we take our focus away from the practical processes and from the activities that people need to carry out to achieve and use knowledge. Their concerns, the tensions they feel, the experience of bringing widely different practices together, all this is being neglected. For these reasons, I think it is crucial to refocus on the practical and material processes of knowledge generation and use.

### 3.1. Not just applying knowledge: What is lost in translation

Diagnosing can hardly be understood just as the application of knowledge, for one thing: “the practice of diagnosing and treating diseases inevitably requires cooperation. […] In the consulting room something is done. […] [T]wo people are required. A doctor and a patient. […] The doctor must ask questions, and the patient must be willing and able to attend to answer them. And in addition to these two people, there are other elements that play a more or less important role. The desk, the chairs, the general practitioner, the letter: they all participate in the events” (Mol, 2002, 22–23).

The cataract is *diagnosable* only in that context. Diagnosing a patient correctly requires many years of training, as well as continuous cooperation with other medical professionals, readings, and trials and errors. It requires interactions with the skill sets of being an optometrist or an ophthalmologist within a specific organization and in local circumstances (Gabbay and Le May, 2010). It requires learning how to handle the conflicting demands of the profession and so much more, such as coordinating with tools, slit lamps, desks and chairs, eye drops, laptops, and databases. A cataract is *diagnosable* just after years of continuously exploring and tuning to the relevant possibilities for action offered by specific settings.

Thus, medical knowledge can hardly be reduced only to textbook information on what a cataract is and the steps to diagnose it. That is because knowledge is not just a decontextualized and universal “product,” but something that is co-constructed and negotiated between different people in different situations. Knowledge emerges over time, as people regularly interact within established practices. It exists only through interaction, and it is embedded in the historical, material, technical, social, and embodied conditions of its production (Haraway, 1988). In other words, knowledge is not static, but rather is always in flux, shaped by the changing conditions of its production, and it is always dependent on institutional, technical, and cultural norms (Kuhn, 1962/1970), on practices that produce it (Pickering, 1992), and on attempts to observe, measure, or comprehend it (Shapin and Schaffer, 1985, Latour, 1987).

Even in the literature on innovation, the importance of contextual factors is widely agreed upon. For example, social influences such as the leadership style (Tierney, 2008; Bledow et al., 2009; Chaman et al., 2021), the team environment, participation, constructive controversy, and intra-group safety are said to be fundamental to innovation (Yakhlef and Rietveld, 2020). But emotions and mood also seem to play a decisive role. Several studies suggested a link between positive mood states and innovation (Amabile et al., 2005; Bledow et al., 2009; Brimhall and Mor Barak, 2018; Yakhlef and Rietveld, 2020; Aslan and Atesoglu, 2021). Interestingly, material and physical aspects are also relevant, including the working space. For instance, it seems that long corridors facilitate a hierarchical organization, with people working in separate offices, while a flat space allows people to interact regardless of their position and rank (Kristensen, 2004).

These findings attribute to sociocultural norms and physical context a significant impact on the way we generate and apply knowledge, and that needs to be taken seriously. In fact, our activities and skills are embedded in our broader socio-cultural context (Hodges and Baron, 1992; Costall, 1995; Ingold, 2000/2001; Heft, 2001; Rietveld, 2008), where context is not understood as a fixed set of surrounding conditions but as a wider dynamical process (Hutchins, 1995). For that reason, I think it is important to argue that these contextual elements are not only significant factors that have an impact on the process but also an integral and essential part of the process itself. Take again as an example the “sociocultural norms” in the KT model proposed by the Canadian Institutes of Health Research [CIHR] (2005). In KT4, it is written that after producing research results, we should “place(ing) research findings in the context of other knowledge and sociocultural norms” (Canadian Institutes of Health Research Knowledge Translation). However, the context is not merely a background or container in which something can be placed; it is neither passive nor static, but rather it is constantly changing.

When one conceives of knowledge as embodied and embedded in real-world practices, as is done by enactive and ecological accounts of cognition, skilled activities, social cooperation, and even emotions, the physical structure of the working space, all gain a prominent role (see Heft, 2001; Ingold, 2011; Colombetti, 2013; van Dijk and Rietveld, 2017; Van Dijk and Myin, 2018; McKinney et al., 2022). The ability to understand what one ought to do and what is “afforded” by the relative environment becomes something that enables both knowledge generation and knowledge use.

Consider the daily work of an ophthalmologist. The ophthalmologist has to deal with different activities, including cataract surgery. Hence, she needs to be constantly skillfully attuned to the norms and concerns of the place she is working in, as well as the procedures, documents, privacy forms, and tools available. Additionally, she needs to be responsive to social cooperative practices with nurses and secretaries who can help organize the surgery appointment and carry it out. People who are collaborating, together with the tools and resources provided by their environments, are engaged in richly scaffolded activities that enable them to master practices that they might not be able to handle independently (Hutchins, 1995). All these practices are nested within the daily hospital organization and cannot be neglected in understanding the practice of diagnosing.

When considering the so-called KTA gap, it is important to focus not only on scientific results and how to transfer them from one side of the gap to the other, but also on the ongoing dynamic activities of the participants involved in real-world settings. This allows us to better understand the other translation process mentioned by Newell et al., namely, the cooperation among different experts. In fact, as already pointed out, in the process of developing the forms to be distributed in various hospitals, a group of different experts, with diverse needs and concerns, different jargons, and training, cooperated and managed to overcome the barriers that divided them. The experts managed not only to produce new knowledge together but also to apply it locally with success.

### 3.2. Translating in cooperative practices

The study by Newell et al. not only showed us how the simple “knowledge transfer” or “best practice” was not a success in the Midlands NHS Trust Hospital project but also showed us why it failed. Their findings strongly suggest that the process of generating knowledge about best practices is, practically speaking, inseparably linked with its application in real-world settings. The authors of the study claimed that exchanging ideas and skills among the people involved in the project, as well as practicing together, was essential to creating new meanings and insights. Understanding the success of the process of the implementation of the new best practice without resorting to the collective cooperation process that led to the reformulation of the cataract diagnostic procedure means cutting out the key elements that allowed this process to take place. Consider as an example how the role of the optometrist was redeveloped by the project. In the Section “2.1 Bridging the gap: An attempt”, the optometrist’s traditional role was explained. She is the first to make the preliminarily diagnosis of the cataract. However, the optometrist is not a medical doctor, so she refers the patient to their general practitioner. The general practitioner then refers the patient to the ophthalmologist, who finally confirms the diagnosis and puts the patient on a waiting list for surgery. With the newly developed practice, optometrists were able to determine from the beginning of the patient’s journey if she needed cataract surgery, rather than waiting until further down the line. This new practice was developed by optometrists, ophthalmologists, and other participants in the project through mutual effort and common activity.

One of the optometrists involved in the project from the beginning explained to the authors that the change in his role allowed him to diagnose and refer patients. However, he also explained that there were times when he needed to speak with the consultant in more detail in order to confirm that a particular patient was actually a good candidate for the cataract operation. Furthermore, optometrists are often treated with “contempt” (Newell et al., 2003) by ophthalmologists, which makes the interaction between the two groups more difficult. However, by working together on the project and sharing their professional skills, the consultants involved learned to respect and trust the optometrists’ competencies. Furthermore, the optometrists working on the project could easily ask for advice from the hospital consultant, due to the close relationships built from working on the same project together. Additionally, the consultant provided regular feedback to the optometrists, so they could adjust their practices and learn over time how to make diagnoses that were up to the consultant’s standards. This is a crucial finding, as it clearly demonstrates that optometrists and consultants were able to coordinate their actions and skills and change their respective practices through regular interactions and negotiations. Newell et al. concluded that this “internal knowledge translation process” among the different experts involved in the team worked precisely because it was intertwined with a process of knowledge generation. Without this collective activity, the knowledge and understanding of the various groups would have remained unconnected and isolated, and the preconceived notions of the barriers among professional competencies would have not been challenged.

If the debate on KT recognized that contextual elements have an impact on the process of transferring knowledge from the lab to the clinic, the same cannot be said about the debate about the KTA gap. In fact, the way the KTA gap is still conceptualized reflects the concrete material and temporally extensive practices and their normative aspects through which knowledge is produced, found, and used. Thus, I claim we should focus on these extended practices, recognizing that the phenomenon of the KTA gap is rooted in and constituted through the several activities carried out by doctors and scientists.

## 4. Beyond the KTA gap and toward a discontinuity of practices

The traditional articulation of the KTA gap interprets the lack of coordination between basic research and clinical practice as a gap between empirical results and their application. But, as we saw in the Section “3 From knowledge as an object to knowing in practice,” if we only consider the problem from the perspective of knowledge transfer, we may overlook potential solutions that could be more effective. Therefore, we need to revise the terms of the problem, shifting the focus from knowledge understood as independent from practical circumstances to the situated practices of knowing. This requires us not only to consider all the aspects mentioned in the Section “3 From knowledge as an object to knowing in practice,” such as social coordination, skills, and context, but also to make them the primary focus. I argue that if we focus on these elements, we can see the KTA gap as a discontinuity of practices, as a discontinuity in the way communities engage with their local contexts. A better explanation of what that entails requires us to side with the “practice turn” in the philosophy of science, which at least since the 1970s situates scientific knowledge in the actual doings carried out by scientists in their real settings (Ankeny et al., 2011; Soler et al., 2014, p. 5–6; Pickering, 1992, p. 1).

### 4.1. The practice turn

It is generally acknowledged that philosophy of science and in general science studies undertook a fundamental shift, which emerged from the criticism of the traditional philosophy of science, considered too idealized and disconnected from how science is actually done (Zhu and Tong, 2020). For instance, in his 1987 work *Science in Action*, Bruno Latour writes of science as a Janus-faced phenomenon. On the “bright side,” we see “ready-made science: established and indubitable scientific facts that refer to natural objects that have always been there” (de Boer et al., 2021), but on the other side, the veiled side, we see science-in-the-making, i.e., the side that “does not know yet” (Latour, 1987) and that through constant transactions among its participants, is constantly made and re-made. Examples of this tendency to focus on “science-in-the-making” can be seen in the work done under the label “philosophy of science in practice” as well as in STS (Science and Technology Studies) and SKK (Sociology of Scientific Knowledge).^5^

In the context of this study, practices are understood as the “open-ended, spatially and temporally dispersed nexus of human activities” (Schatzki, 2012). They are open-ended because they are not fixed by a specific and settled number of practices. Practices are in fact continuously and dynamically re-adapted and negotiated by practitioners. Moreover, the activities that compose a practice are spatially and temporally distributed, meaning they are carried out in concrete spaces, such as an ambulatory or laboratory, and occur on different time scales. For example, the practice of diagnosing is spread across several temporal scales, such as the time it takes to conduct the tests, the time it takes to cure a specific disease, and the time it takes to make appointments in a clinic. These activities are not clearly separate and distinct. They are indeed a “nexus” because they “hang together” (Schatzki, 2012).

If we adopt this perspective, the frame of the problem changes. Instead of being focused on knowledge and how to transport it, it extends beyond it to include practices and real-world settings, socio-cultural norms, tools, skills, etc. This expands the range of relevant elements and re-situates the gap in the activities of both scientists and doctors, creating a new understanding of the problem.

### 4.2. Misalignment of practices: From a gap to a discontinuity

If we focus on the actual settings and seriously consider the material, social, and cognitive aspects of the practices, we can see the KTA gap as a discontinuity in the way communities engage with their local contexts and in what they actually perceive as appropriate for their activities. The communities involved can be understood as coping with different environments and necessities, performing different tasks, and developing different ways to carry them out; their apparent incommensurability comes from the necessity to cope with different situations. The reason to use the term “discontinuity” instead of “gap” is to more accurately describe the misalignment of their practices and the material and social difficulties and tensions that the communities experience in interacting. But more importantly, to highlight that there is indeed a common ground, they are not substantially different. Take again the cooperation process experienced by the project participants at the Midlands NHS Trust Hospital as an example. The project conceived by the NHS has brought together several experts who, although part of different processes in the diagnosis of a cataract, rarely come into contact. Each of these experts practices different things and has different “ends-in-view.” The optometrist usually diagnoses eye problems to prescribe glasses. The general practitioner determines if it is necessary to refer the patient to a specialist. The ophthalmologist instead attempts to cure the disease through specific drugs or surgery. Each of these practices is intertwined with different settings, norms, and ways of coordinating with the environment. When these practices entangled with the aim of creating a new procedure that would have involved them all, the misalignment between these disciplines and activities blurred and reconfigured as they were collaborating.

But indeed, the practices at the beginning were not coordinated. One of the most disputed changes in the new cataract practice, for example, was the role of the consultant’s secretary (Newell et al., 2003). Under the old model, each secretary was responsible for all operating theater scheduling for one designated consultant surgeon. Under the new practice, an administrative assistant carried out all theater planning, and secretaries were redistributed to more than one consultant. The secretaries, who insisted they were far too busy to be assigned to more than one consultant, were “extremely” resistant to this change, as “they saw the waiting list management as a big part of their role. They felt that we were undermining their role by taking this away…taking away their patient contact…we were just turning them into audio typists (Project Member)” (Newell et al., 2003). The new practice did not seem right to the secretaries. They felt that something constitutive of their practice had been taken away. Even after various attempts to improve the relationships between the secretaries and the administrative assistant, the secretaries continued to express reluctance toward the new practice. For example, when the new administrator in charge of operating theater scheduling started, she was not given the theater schedules from the individual consultants’ secretaries. As a result, she was not able to do her job properly. “They were wanting her to sink (Project Member)” (Newell et al., 2003).

The new practice and the secretaries’ nexus of activities were not well-aligned. The secretaries felt from the beginning that something was wrong and that resistance to the new practice was the result. The difficulties and tensions cannot be adequately explained from the perspective of the KTA gap, which could see this problem as a gap between knowledge of best practices and their implementation. However, if we do not take into account the secretaries’ practices and the normative tensions between them, the project, and the assistant, it is impossible to understand why the new practice was not adopted, as the relevant processes that contributed to its non-use were not connected to knowledge but to the elements and processes described in the Section “3 From knowledge as an object to knowing in practice.”

When different communities have misaligned practices, it creates a coordination problem, which often happens between basic science and clinical practice. For example, it is said that, unlike scientists, “to a clinician, asking “why?” distracts from the sense of mastery that comes from accumulating information and applying it in a clinical setting” (Restifo and Phelan, 2011). Or that the dynamics of the basic biomedical research field feed on “promotions and grants based largely on the papers scientists have published in top journals, not on how much they have advanced medicine” (Butler, 2008).

The fact that there are these cultural misalignments and major communication difficulties does not mean, however, that there is no common ground. For this reason, I believe that revising the problem not as a gap between knowledge and practice but as a discontinuity in the way people are skillfully coping and practicing is a better perspective. First, it takes into account the practical and distributed processes of knowledge generation and use. Second, it takes seriously the difficulties and sociocultural barriers among these communities. Third, it provides a common ground on which to look for new solutions.

If we focus on the fact that both scientists and clinicians are attuned to different practices and are skillfully responding to their local environments, we can expect that if the practices are shared, such as in the case study by Newell et al., a new order of self-organizing responses and activities will emerge. Knowledge translation then could involve reconfiguring practices and experience: learning to participate in several different doings and changing their criteria for success together as these practices get coordinated over time. For this reason, we should pay attention to how this reconfiguration takes place in practice, as it can be the starting point for redefining knowledge translation.

I suggest that as a next step to reformulate knowledge translation, we should tighten the connection between ecological psychology and enactivism, and STS. That first connection, already established by different scholars (Ingold, 2011; Rouse, 2018; Abrahamson et al., 2019; Van Dijk and Myin, 2019; Zhu and Tong, 2020; Froese, 2022), and foregrounded by this paper, needs to be considered more attentively. In the context of revising knowledge translation, ecological psychology, and enactivism could focus on the experiential and normative aspects of coordination among different practices, while STS could provide the methods for studying the practices themselves, e.g., through ethnography and qualitative research.

## 5. Conclusion

The study aimed to be a proposal, a revision of a problem, and an invitation to consider the practical, extended, and distributed processes involved in knowledge generation and knowledge use. Scientific and clinical practices involve non-linear interactions and synergies between materials, social practices, and people skillfully responding and interacting with different environments and settings. By conceptualizing a problem by neglecting these aspects or making them secondary, means constraining the terrain on which solutions can be sought. Therefore, it is partial to state that the lack of communication between those who conduct basic scientific research and those who provide clinical care is simply a gap between research results and practical application. Instead, this disconnect should be seen in terms of a discontinuity of practices.

In the Section “1 Introduction”, I explained that the KTA gap is a metaphor that frames the problem of the lack of coordination between basic science and clinical practice in terms of a gap between empirical results and practical application. In the Section 2” From the KTA gap to knowledge translation” instead, I have focused on why it is important to reformulate the terms of the problem, and I have claimed that it is because the KTA gap frames the solutions around certain unquestioned assumptions that reify knowledge, limiting the effectiveness of KT itself. In the Section “3 From knowledge as an object to knowing in practice,” I have focused on why it is important to go beyond reification, and what it leaves out. I have claimed that reification brings us away from the practical processes of knowledge generation and use, skills, context, cooperative practices, tools, and socio-cultural norms. In the Section “4 Beyond the KTA gap and toward a discontinuity of practices”, I have claimed that if we foreground these elements and look at real-world practices, we do not see gaps but a discontinuity in the way people cope with their surroundings.

Reformulating the problem and opening it up to practices is only the beginning. The next step is to re-articulate this new perspective in practice. This means exploring the connection between ecological psychology and enactivism, and STS, where ecological psychology and enactivism could focus on the experiential and normative aspects of coordination among different practices, as already happens in the context of other kinds of practices, such as architecture (Rietveld and Kiverstein, 2014; Rietveld and Brouwers, 2017; van Dijk and Rietveld, 2017, 2021), pretend play (Szokolszky and Read, 2021; Murphy, 2022), education (Young, 2004; Gresalfi et al., 2012; Phillips and Finn, 2022), and many others. On the contrary, STS could provide the methods for studying the practices themselves, e.g., through ethnography and qualitative research (Latour and Woolgar, 1979; Latour, 1987; Mol, 2002; Greiffenhagen et al., 2011). For that reason, I would encourage an in-depth look at this convergence, as it could potentially lead to finding new solutions, but it could also help in clarifying other factors, such as how the experiential and normative aspects of scientific practices are coordinated in real-world practices.

## Data availability statement

The original contributions presented in this study are included in the article/supplementary material, further inquiries can be directed to the corresponding author.

## Author contributions

GD wrote the manuscript, developed the argument, contributed to the article, and approved the submitted version.

## Footnotes

1 It is frequently stated that it takes an average of 17 years for research evidence to reach clinical practice. For instance, Balas and Boren (2000) and Grant et al. (2003) estimated a time lag of 17 years when measuring different points of the process. On the one hand, Balas and Boren (2000) calculated the gap in advancements in diabetic eye care, while on the other hand, Grant et al. (2003) calculated the gap in advances in neonatal intensive care. Grant et al. determined it through a bibliometric analysis. They claimed in fact that “by looking at the journals in which papers cited on clinical advances are published, it is possible to characterize the research and estimate how long it takes for basic research to feed into clinical practice.” However, even if there is a general consensus on the 17-year time lag, measuring what is actually a “time lag” is a difficult task, as Hanney et al. (2015) demonstrated.

2 Results of studies conducted in the United States and the Netherlands suggest that most of the time, guidelines are not applied: “about 30–40% of patients do not receive care according to present scientific evidence” (Grol and Grimshaw, 2003). However, these percentages are problematic, as they are already based on normative assumptions, such as the fact that cure programs need to be evidence-based to be beneficial. Despite this, I think it is important to name them as the present literature discusses them as a knowledge-to-action gap to be resolved and the solutions sought are discussed on the basis of these formulations.

3 PAHO. Knowledge Translation for Health Decision Making. Accessed on 2022/07/5. Available from: https://www3.paho.org/hq/index.php?option=com_content&view=article&id=9682:knowledge-translation-for-health-decision-making&Itemid=41010&lang=en.

4 See, for example, “Understanding-User-Context Framework” (Jacobson et al., 2003); “The Ottawa Model of Research Use” (Logan and Graham, 1998); “The Knowledge-to-Action Process Framework” (Graham et al., 2006); and “The Promoting Action on Research Implementation in Health Services Framework” (Kitson et al., 1998; Rycroft-Malone et al., 2002, 2004).

5 The Society for Philosophy of Science in Practice (SPSP) grew out right from this tendency, as they recognized the need to promote the philosophical study of “science in practice.”
